# Complete Genome Sequence of Ehrlichia canis Strain YZ-1, Isolated from a Beagle with Fever and Thrombocytopenia

**DOI:** 10.1128/genomeA.00133-18

**Published:** 2018-03-01

**Authors:** Jilei Zhang, Jiawei Wang, Chengming Wang

**Affiliations:** a College of Veterinary Medicine, Yangzhou University, Yangzhou, Jiangsu, China; b College of Veterinary Medicine, Auburn University, Auburn, Alabama, USA

## Abstract

We report the complete genome sequence of Ehrlichia canis strain YZ-1, which was isolated from a beagle with fever, anorexia, depression, lethargy, weight loss, and thrombocytopenia. E. canis is the tick-borne agent of canine and human monocytic ehrlichiosis.

## GENOME ANNOUNCEMENT

Ehrlichia species, which are important tick-borne pathogens in humans and animals, are obligate intracellular Gram-negative bacteria of the family Anaplasmataceae. Five Ehrlichia species are recognized, namely E. chaffeensis, E. ewingii, E. canis, E. ruminantium, and E. muris, with three (E. canis, E. chaffeensis, and E. ewingii) causing canine ehrlichiosis (1–3). Complete genome sequences have previously been reported for E. chaffeensis strain Arkansas (4), E. muris strain AS145 (5), E. canis strain Jake (6), three strains of E. ruminantium (7), and E. mineirensis, a novel organism closely related to E. canis (8). Here, we describe the second complete genome sequence of E. canis, made from strain YZ-1, which was isolated from an adult female beagle (Canis lupus familiaris) living in a commercial dog farm in Taizhou, Jiangsu, China. The dog was clinically ill, with signs that included fever (41.4°C), depression, lethargy, anorexia, and thrombocytopenia (1.2 × 10^10^ platelets/liter).

The E. canis YZ-1 strain isolate has been maintained in our laboratory by continuous passage in the DH82 canine monocytic cell line (ATCC CRL-10389) for 20 passages. For genome sequencing, the organisms were extracted from DH82 cells and purified by Percoll density-gradient centrifugation. The E. canis YZ-1 DNA was extracted using the Qiagen DNA minikit (Qiagen, Valencia, CA) and a 1-μl aliquot quantified using the Qubit fluorometric quantification system (Thermo Fisher Scientific, Waltham, MA). The genome of E. canis strain YZ-1 was sequenced by single molecule real-time (SMRT) technology using PacBio systems (Pacific Biosciences, USA) at Beijing Novogene Bioinformatics Technology Co., Ltd. (Beijing, China). A total of 146,443 pass-filter quality reads of 1.8 × 10^9^ bp in length were generated, showing an average read score of 84%. The *de novo* assembly of the reads using SMRT Portal resulted in 156 polished contigs and 2,735,875 bases, with an *N*_50_ value of 129,950. A single contig status without gaps was achieved for the genome, and the circular chromosomal structure was validated.

The genome of E. canis strain YZ-1 consists of a single circular chromosome containing 1,314,789 nucleotides with an overall G+C content of 29%. The origin of replication (*oriC*) was predicted using the Ori-Finder program to be a 425-nucleotide region upstream of a hypothetical protein (locus tag EC-YZGM000006), and 1 bp was assigned to be the beginning of the *oriC* (9). The genome was annotated using the NCBI Prokaryotic Genome Annotation Pipeline version 4.3, which used GeneMarkS+ for gene prediction (https://www.ncbi.nlm.nih.gov/genome/annotation_prok/). The genome of E. canis YZ-1 is predicted to contain 994 genes, including 924 protein-coding sequences (CDSs), one copy of each of the rRNA genes (5S, 16S, and 23S), 36 tRNA genes, 29 pseudogenes, and two small noncoding RNAs. The availability of the complete genome of E. canis YZ-1 will enable divergence with other Ehrlichia spp. to be determined and studied and enable more reliable insights into the evolution of the species and mechanisms of their host specificity.

### Accession number(s).

The E. canis strain YZ-1 whole-genome sequence has been deposited in GenBank under the accession number CP025749.

## References

[1] Dumler JS, Barbet AF, Bekker CP, Dasch GA, Palmer GH, Ray SC, Rikihisa Y, Rurangirwa FR 2001 Reorganization of genera in the families *Rickettsiaceae* and *Anaplasmataceae* in the order *Rickettsiales*: unification of some species of *Ehrlichia* with *Anaplasma*, *Cowdria* with *Ehrlichia* and *Ehrlichia* with *Neorickettsia*, descriptions of six new species combinations and designation of *Ehrlichia equi* and 'HGE agent' as subjective synonyms of *Ehrlichia phagocytophila*. Int J Syst Evol Microbiol 51:2145–2165. doi:10.1099/00207713-51-6-2145. 11760958

[2] McBride JW, Walker DH 2011 Molecular and cellular pathobiology of *Ehrlichia* infection: targets for new therapeutics and immunomodulation strategies. Expert Rev Mol Med 13:e3. doi:10.1017/S1462399410001730. 21276277 PMC3767467

[3] Zhang J, Kelly P, Guo W, Xu C, Wei L, Jongejan F, Loftis A, Wang C 2015 Development of a generic *Ehrlichia* FRET-qPCR and investigation of ehrlichiosis in domestic ruminants on five Caribbean islands. Parasit Vectors 8:506. doi:10.1186/s13071-015-1118-5. 26438311 PMC4595018

[4] Dunning Hotopp JC, Lin M, Madupu R, Crabtree J, Angiuoli SV, Eisen JA, Eisen J, Seshadri R, Ren Q, Wu M, Utterback TR, Smith S, Lewis M, Khouri H, Zhang C, Niu H, Lin Q, Ohashi N, Zhi N, Nelson W, Brinkac LM, Dodson RJ, Rosovitz MJ, Sundaram J, Daugherty SC, Davidsen T, Durkin AS, Gwinn M, Haft DH, Selengut JD, Sullivan SA, Zafar N, Zhou L, Benahmed F, Forberger H, Halpin R, Mulligan S, Robinson J, White O, Rikihisa Y, Tettelin H 2006 Comparative genomics of emerging human ehrlichiosis agents. PLoS Genet 2:e21. doi:10.1371/journal.pgen.0020021. 16482227 PMC1366493

[5] Thirumalapura NR, Qin X, Kuriakose JA, Walker DH 2014 Complete genome sequence of *Ehrlichia muris* strain as145^T^, a model monocytotropic *Ehrlichia* strain. Genome Announc 2:e01234-13. doi:10.1128/genomeA.01234-13. PMC3907729 24482514

[6] Mavromatis K, Doyle CK, Lykidis A, Ivanova N, Francino MP, Chain P, Shin M, Malfatti S, Larimer F, Copeland A, Detter JC, Land M, Richardson PM, Yu XJ, Walker DH, McBride JW, Kyrpides NC 2006 The genome of the obligately intracellular bacterium *Ehrlichia canis* reveals themes of complex membrane structure and immune evasion strategies. J Bacteriol 188:4015–4023. doi:10.1128/JB.01837-05. 16707693 PMC1482910

[7] Frutos R, Viari A, Ferraz C, Morgat A, Eychenié S, Kandassamy Y, Chantal I, Bensaid A, Coissac E, Vachiery N, Demaille J, Martinez D 2006 Comparative genomic analysis of three strains of *Ehrlichia ruminantium* reveals an active process of genome size plasticity. J Bacteriol 188:2533–2542. doi:10.1128/JB.188.7.2533-2542.2006. 16547041 PMC1428390

[8] Cabezas-Cruz A, Zweygarth E, Broniszweska M, Passos LM, Ribeiro MF, Manrique M, Tobes R, de la Fuente J 2015 Complete genome sequence of *Ehrlichia mineirensis*, a novel organism closely related to *Ehrlichia canis* with a new host association. Genome Announc 3:e01450-14. doi:10.1128/genomeA.01450-14. PMC4319577 25614563

[9] Gao F, Zhang CT 2008 Ori-finder: a web-based system for finding *oriC*s in unannotated bacterial genomes. BMC Bioinformatics 9:79. doi:10.1186/1471-2105-9-79. 18237442 PMC2275245

